# Supplementary material for: Biomechanical performance of resin composite on dental tissue restoration: A finite element analysis
Source: PLoS One. 2023 Dec 21;18(12):e0295582. doi: 10.1371/journal.pone.0295582 (PMC10734934; doi:10.1371/journal.pone.0295582)
Supplement: Attachment — Submitted filename: Comments_R2_16-11-2023.docx [file pone.0295582.s003.docx]

**Comments to the Author**

1. If the authors have adequately addressed your comments raised in a previous round of review and you feel that this manuscript is now acceptable for publication, you may indicate that here to bypass the “Comments to the Author” section, enter your conflict of interest statement in the “Confidential to Editor” section, and submit your "Accept" recommendation.

Reviewer #1: All comments have been addressed

Reviewer #2: All comments have been addressed

Reviewer #3: All comments have been addressed

2. Is the manuscript technically sound, and do the data support the conclusions?

The manuscript must describe a technically sound piece of scientific research with data that supports the conclusions. Experiments must have been conducted rigorously, with appropriate controls, replication, and sample sizes. The conclusions must be drawn appropriately based on the data presented.

Reviewer #1: Yes

Reviewer #2: No

Reviewer #3: Yes

3. Has the statistical analysis been performed appropriately and rigorously?

Reviewer #1: N/A

Reviewer #2: Yes

Reviewer #3: Yes

4. Have the authors made all data underlying the findings in their manuscript fully available?

The [PLOS Data policy](http://www.plosone.org/static/policies.action#sharing) requires authors to make all data underlying the findings described in their manuscript fully available without restriction, with rare exception (please refer to the Data Availability Statement in the manuscript PDF file). The data should be provided as part of the manuscript or its supporting information, or deposited to a public repository. For example, in addition to summary statistics, the data points behind means, medians and variance measures should be available. If there are restrictions on publicly sharing data—e.g. participant privacy or use of data from a third party—those must be specified.

Reviewer #1: Yes

Reviewer #2: Yes

Reviewer #3: Yes

5. Is the manuscript presented in an intelligible fashion and written in standard English?

PLOS ONE does not copyedit accepted manuscripts, so the language in submitted articles must be clear, correct, and unambiguous. Any typographical or grammatical errors should be corrected at revision, so please note any specific errors here.

Reviewer #1: Yes

Reviewer #2: Yes

Reviewer #3: Yes

6. Review Comments to the Author

Please use the space provided to explain your answers to the questions above. You may also include additional comments for the author, including concerns about dual publication, research ethics, or publication ethics. (Please upload your review as an attachment if it exceeds 20,000 characters)

**Reviewer #1:**

I am thoroughly satisfied with the current version as it fulfills all my requirements and expectations to the fullest extent of this paper.

🡪**Response:**

Thank you for accepting the revised version; I appreciate your approval for the article's publication in the journal.

**Reviewer #2:**

With the help of the reviewers, this revised and re-submitted manuscript has been considerably improved. Notwithstanding, still some minor issues would seem in need of revision, please see below.

1- “The first hypothesis posits that (...)" and "The second hypothesis suggests that (...)" - please provide a sound and valid NULL HYPOTHESIS. The latter must be deducible from the forgoing thoughts.

🡪 **Response:**

Thank you for your valuable suggestions to enhance the manuscript. The introduction has been revised incorporating the following paragraph:

“The first null hypothesis maintains that no notable disparity exists in stress distribution within enamel and dentin between an infected tooth and a restored or healthy tooth. The second null hypothesis contends that the stiffness of restorative materials holds no consequential influence on the stress distribution observed in restored teeth.”

2- “The results revealed that the first hypothesis, which proposed that there would be a significant difference in stress distribution between infected, healthy, and restored teeth, was true." Please note that a hypothesis never will be "true". Again, please provide a sound NULL hypothesis (see comments given above), and remember that H0 can be rejected or not rejected.

🡪 **Response:**

Thanks for your comments to improve the manuscript. We have revised carefully as suggested by reviewer.

The results revealed that the first null hypothesis, suggesting no significant difference in stress distribution between infected, healthy, and restored teeth, was not supported and was consequently rejected.

3- Same with "The second hypothesis of the study (...) was also true." Revise carefully.

🡪 **Response:**

The study's findings led to the rejection of both null hypotheses. Thank you for your valuable insights.

“The second null hypothesis, which stated that the stiffness of restorative materials has no consequential influence on the stress distribution observed in restored teeth, was also not supported and therefore was rejected.”

4- "This highlights the importance of early detection and treatment of dental infections to prevent the progression of damage and the need for extensive restorative procedures." Sound references missing. Again, please stick to <https://pubmed.ncbi.nlm.nih.gov/19639091/>, to <https://doi.org/10.13140/RG.2.2.36646.37443>, and to <https://pubmed.ncbi.nlm.nih.gov/28294198/>. Please discuss.

🡪 **Response:**

We appreciate your valuable input in enhancing the manuscript. Following your guidance, the references mentioned above have been incorporated to strengthen and improve the manuscript.

“Addressing early detection and treatment of dental infections promptly becomes pivotal in preventing the progression of damage and the subsequent necessity for extensive restorative techniques. Research investigating resin infiltration techniques [3,43,44] substantiates this notion, demonstrating their capacity to halt the advancement of white spot lesions. This underscores the importance of timely intervention as a preventive measure against the need for extensive dental restorations”.

3.	Kielbassa AM, Muller J, Gernhardt CR. Closing the gap between oral hygiene and minimally invasive dentistry: a review on the resin infiltration technique of incipient (proximal) enamel lesions. Quintessence Int. 2009;40: 663–81.

43.	Kielbassa AM, Ulrich I, Werth VD, Schüller C, Frank W, Schmidl R. External and internal resin infiltration of natural proximal subsurface caries lesions: A valuable enhancement of the internal tunnel restoration. Quintessence Int (Berl). 2017;48.

44.	Kielbassa AM, Ulrich I, Treven L, Mueller J. An updated review on the resin infiltration technique of incipient proximal enamel lesions. Med Evol. 2010;16: 3–15.

- "An in vitro study reported that the use of composite resin reduces the amount of stress concentration at the interface between the adhesive and the tooth in comparison with other materials that have a higher Young modulus than enamel [42]." Remember that one sentence does not constitute one paragraph. Please provide digestible units.

🡪 **Response:**

We appreciate your guidance to enhance the manuscript; the paragraph below has been revised accordingly

“Composite resins, designed to replicate the natural appearance of teeth and closely mimic their mechanical properties, demonstrate a favorable attribute in adapting to the natural flexure and movement of teeth during function. This adaptability potentially contributes to a more uniform distribution of stress forces, mitigating the likelihood of stress concentration points at the adhesive-tooth interface. An in vitro study corroborated this behavior, demonstrating that the use of composite resin reduces stress concentration at the interface between the adhesive and the tooth compared to materials with a higher Young's modulus than enamel [42].”

**Reviewer#3:**

Reviewer #3: Although there are points about the number of elements, convergence study and quadratic element that I disagree with the author, I think that it is acceptable due to relative comparisons within itself. Other explanations and arrangements are sufficient and I think that the article can be published in your journal.

🡪**Response:**

Thank you for approving the revisions and for your recommendation to publish the article in the journal.

**The authors thank all the reviewers for their valuable comments to improve the paper quality.**
